# The effect of FMT and vitamin C on immunity-related genes in antibiotic-induced dysbiosis in mice

**DOI:** 10.7717/peerj.15356

**Published:** 2023-05-11

**Authors:** Xiaorong Huang, Yv Zhang, Junsong Huang, Wenli Gao, Xie Yongfang, Chuisheng Zeng, Chao Gao

**Affiliations:** 1 Chongqing Key Laboratory of Big Data for Bio Intelligence, Chongqing University of Posts and Tele-communications, Chongqing, China; 2 University of Chinese Academy of Sciences, Beijing, China; 3 Chongqing University of Posts and Telecommunications, Chongqing Key Laboratory of Big Data for Bio Intelligence, Chongqing, China

**Keywords:** Antibiotics, Intestinal microbiome, Fecal microbiota transplantation, Liver, Intestine, Vitamin C

## Abstract

Antibiotics are double-edged swords. Although antibiotics are used to inhibit pathogenic bacteria, they also run the risk of destroying some of the healthy bacteria in our bodies. We examined the effect of penicillin on the organism through a microarray dataset, after which 12 genes related to immuno-inflammatory pathways were selected by reading the literature and validated using neomycin and ampicillin. The expression of genes was measured using qRT-PCR. Several genes were significantly overexpressed in antibiotic-treated mice, including CD74 and SAA2 in intestinal tissues that remained extremely expressed after natural recovery. Moreover, transplantation of fecal microbiota from healthy mice to antibiotic-treated mice was made, where GZMB, CD3G, H2-AA, PSMB9, CD74, and SAA1 were greatly expressed; however, SAA2 was downregulated and normal expression was restored, and in liver tissue, SAA1, SAA2, SAA3 were extremely expressed. After the addition of vitamin C, which has positive effects in several aspects, to the fecal microbiota transplantation, in the intestinal tissues, the genes that were highly expressed after the fecal microbiota transplantation effectively reduced their expression, and the unaffected genes remained normally expressed, but the CD74 gene remained highly expressed. In liver tissues, normally expressed genes were not affected, but the expression of SAA1 was reduced and the expression of SAA3 was increased. In other words, fecal microbiota transplantation did not necessarily bring about a positive effect of gene expression restoration, but the addition of vitamin C effectively reduced the effects of fecal microbiota transplantation and regulated the balance of the immune system.

## Introduction

Antibiotics (ABX) are double-edged swords. Although antibiotics are used to inhibit pathogenic bacteria, they also run the risk of destroying some of the healthy bacteria in our bodies. The intestinal microbiota attaches to and colonizes the gut and participates in intestinal food processing and host nutrition. The intestinal microbiota also promote the maturation of the intestinal epithelium and maintains the integrity of the body’s immune system and natural barrier function (Dave et al., 2012; Rashidi et al., 2021). Similarly, when broad-spectrum antibiotics are administered orally, they can cause dysbiosis of the intestinal microbiota (Yao et al., 2016).

After antibiotic treatment, major defects in the production and activation of innate immune cells are demonstrated. The dysbiotic microbiota then translocates to colonization sites in the gut, which can lead to chronic periepithelial inflammation with barrier leakage, triggering a local inflammatory response or systemic immunity (Levy et al., 2017).

Fecal microbiota transplantation (FMT) is a gut therapy in which bacteria from the feces of a healthy donor are transferred to the patient *via* a different route of administration, thereby restoring the microbial ecosystem in the gut (Li et al., 2016; Yadav et al., 2016). FMT has been found to reverse damaged intestinal microbiota receiving antibiotics, and an additional significant increase in species with anti-inflammatory properties was found in the intestinal microbiota of mice receiving FMT (Le Bastard et al., 2018). FMT is also used for the treatment of inflammatory bowel disease (IBD) but is still not the best choice because of the uncertainty of harmful intestinal microorganisms, low remission rates, variable response to treatment, difficulty in donor selection, and long-term short-term safety, among other reasons (Stojek, Jablonska & Adrych, 2021).

Vitamin C, a micronutrient, is a recognized antioxidant. Vitamin C deficiency is also commonly found in patients with IBD (Dunleavy et al., 2021). Also, high doses of vitamin C have been found to reduce lung inflammation (Jeong et al., 2010) and selectively affect the production of cytokines in the cytoplasm, including the IL-6-producing monocytes, TNF-α, and the number of IL-2-producing lymphocytes, which are important for the immune system (Hartel et al., 2004); therefore, vitamin C has a potential role for immunomodulation. It has also been found that vitamin C has a role in the anti-inflammatory effects of the gastrointestinal tract. Ascorbic acid prevents *Helicobacter pylori* infection and gastric cancer by reactivating vitamin E and glutathione through its cytoprotective antioxidant effect in the stomach and by inhibiting endogenous N-nitrosylation and reducing the toxic effects of ingested nitrosodimethylamine and heterocyclic amines (Toh & Wilson, 2020). Also, vitamin C alleviated acute small intestinal colitis in Campylobacter jejuni-infected mice, and treatment not only attenuated the macroscopic sequelae of infection, but also inhibited apoptosis and inflammatory immune cell responses in the intestine, accompanied by insignificant pro-inflammatory cytokine secretion (Mousavi et al., 2020).

The gut-liver axis refers to the bidirectional relationship between the gut and its microbiota and the liver. Dysbiosis of the microbiota leads to disruption of the gut barrier function, and various microbial-related harmful factors enter the liver along the gut-liver axis. Components of the microbiota or related metabolites can mediate the activation of relevant signaling pathways and play an important role in the liver (Luo et al., 2022). Therefore, the search for key signaling molecules between the gut and the liver is of considerable significance for the selection of specific antibiotics and the specification of FMT.

Given that microbial dysbiosis occurs after ABX treatment, we sought to determine whether FMT would be a safe and efficacious method of modifying the bacterial community. We hope to understand whether FMT alters the immune profile of the gut and liver, which are essential to prevent the harmful consequences of using FMT as a treatment in the future. This study established a gut dysbiosis mouse model by oral intake of broad-spectrum antibiotics, which activates the inflammatory and immune signaling pathway. The data suggest that FMT may enhance gastrointestinal integrity by enhancing immunity. Interestingly, while FMT tended to improve intestinal health, FTM treatment appeared to be more inflammatory than saline controls, the increased expression of pro-inflammatory adaptive immune-related genes suggests a potential response of intestinal and hepatocytes to the altered microbiota induced by FMT.

## Material and Methods

### GEO dataset

**Figure 1:**
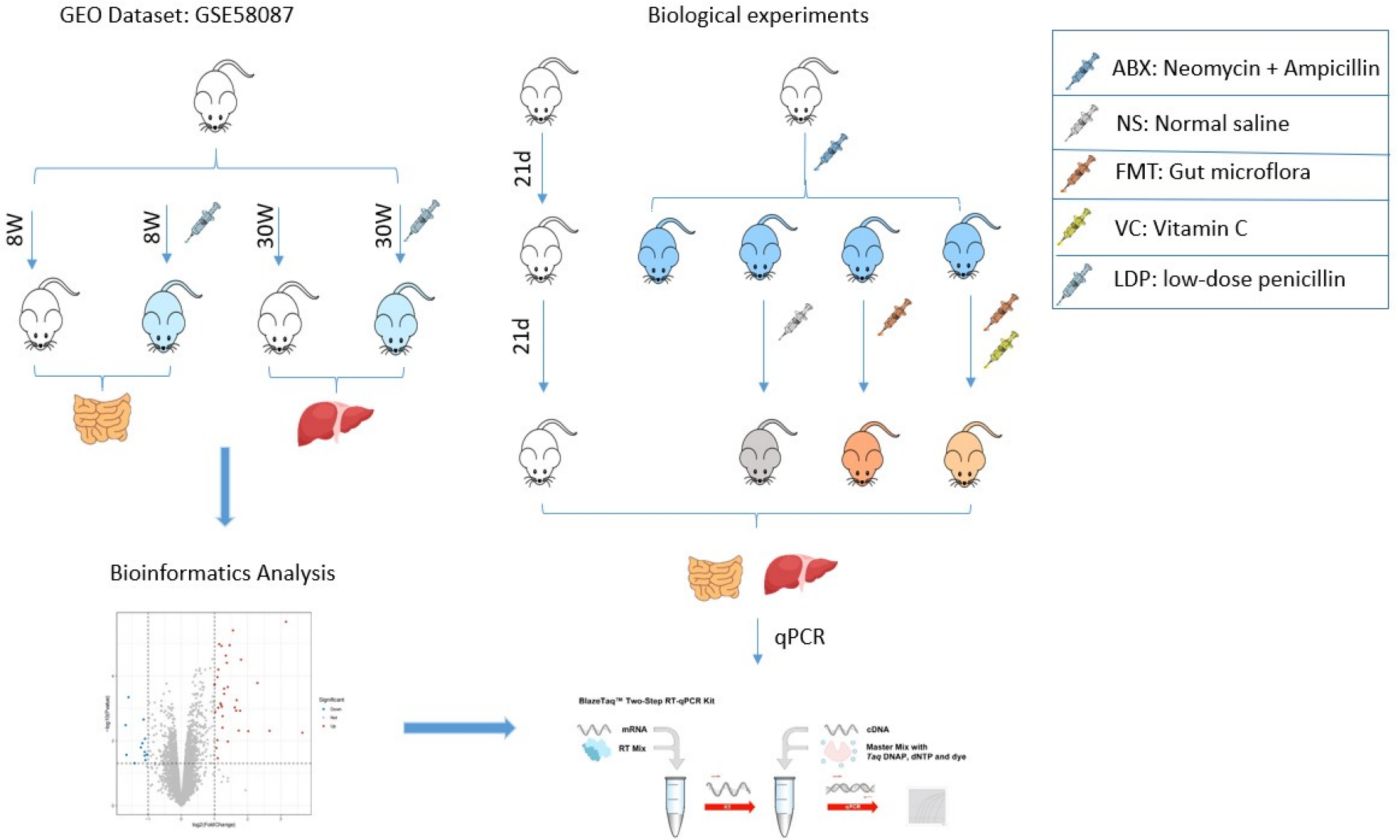
Research flow chart.

The Gene Expression Omnibus (GEO) database is a gene expression database created and maintained by the National Center for Biotechnology Information NCBI. We selected the data set with the registration number GSE58087 to analyze the effect of antibiotics on the organism. The data set has several samples, and we selected four groups of mice with normal ileum for 8 weeks, low-dose penicillin for 8 weeks ileum, normal week liver for 30 weeks, and low-dose penicillin for 30 weeks liver for differential gene expression analysis and functional enrichment analysis (Fig. 1).

### Animals

Fifteen 4-week-old C57BL/6J male mice (15∼25g) were purchased from SPF(Beijing) BIOTECHNOLOGY Co., Ltd. and housed individually in an experimental environment at 23 °C with a 12-hour light/dark cycle. During the experiment, mice had free access to sterilized food and water. After 10 days of acclimation, three mice were used as a control group (NO-ABX) for group N. Twelve mice received antibiotics for 21 days and were later randomly divided into four groups of three mice each in a cage for groups ABX, F, VF, and NS. The ABX group was executed by cervical dislocation after receiving antibiotics for 21 days, and intestinal and liver tissue samples were frozen in liquid nitrogen and stored at −80 °C. The NS group received antibiotics for 21 days followed by saline for 21 days, while the F group received fecal microbial transplantation for 21 days and the VF group received fecal microbial transplantation and vitamin C for 21 days. On day 42 of the experiment, mice in the N, F, VF, and NS groups were executed by cervical dislocation, and intestinal and liver tissue samples were frozen in liquid nitrogen and stored at −80 °C. The collected tissue samples were subjected to quantitative real-time PCR (Fig. 1). All animals were cervically dislocated for sample collection, and no residual animals remained after the experiment.

The Research Ethics Committee of Chongqing University of Posts and Telecommunications approved all experimental procedures (approval reference numbers: 20210324-1).

### Antibiotic therapy and fecal microbiota transplantation

Single antibiotics have limited clearance efficiency for complex microbial ecosystems (Gopalakrishnan et al., 2021). Christopher Staley’s study showed that an extended course of antibiotics better disrupted the original intestinal microbiota in mice (Staley et al., 2017), while Vancheswaran Gopalakrishnan found that a 3-week antibiotic regimen better promoted microbial community transformation and better acceptance of donor flora (Gopalakrishnan et al., 2021). After ten days of self-adaptation, we selected neomycin (0.5 mg/ml), which has a small degree of systemic absorption, and ampicillin (1 mg/ml), which has a large degree of systemic absorption, for antibiotic treatment of mice (Lynn et al., 2021; Staley et al., 2017). It was dissolved in sterile drinking water and filtered, obtained *ad libitum* by the mice, and changed once a day for 21 days of administration.

The role of FMT is also limited in that it allows the establishment of only a small fraction of the human bacterial community (Wos-Oxley et al., 2012). Korry J Hintze suggested that two to three weeks after antibiotic treatment is the best time for fecal microbiota transplantation (Hintze et al., 2014), and for better retention of the donor mouse flora in the recipient mice, we chose to transplant mice with fecal microbiota for 21 consecutive days. Fecal microbiota transplantation (FMT) was performed using samples made from untreated feces of healthy mice. The feces of healthy mice were mixed with sterile saline (40 mg/ml) and homogenized. After homogenization, centrifugation (800g, 3min, 4 °C) was performed and the supernatant was collected, which was the transplantation sample. The treated mice were given 100uL of supernatant by gavage for 21 consecutive days, and an additional 100ul of 200mg/kg VC solution was given to mice in group 4. Group 2 mice recovered naturally and were given only 200ul of sterile saline, and normal mice in Group 5 were given sterile food and quoted water as normal (Fig. 1).

### Quantitative Real-time PCR

Total RNA was extracted from collected intestinal and liver tissues using TRIzol reagent (Invitrogen; Thermo Fisher Scientific, Inc., Waltham, MA, USA) according to the manufacturer’s protocol, and the purity of RNA was measured using spectrophotometric analysis (Quawell Q3000, USA). Next, RNA was reverse transcribed to cDNA using the RevertAid First Strand cDNA Synthesis Kit (Thermo Scientific, Waltham, MA, USA). The quantitative real-time PCR (qRT-PCR) analysis was performed using SYBRPRIME qPCR Kit (Baoguang Biotechnology Co., Ltd., Chengdu, China) and Bio-Rad iQ5 software (Bio-Rad, Hercules, CA, USA). GAPDH was analyzed in each sample to normalize expression. Three biological replicates were performed for each gene, and each replicate contained three technical replicates. The primers used in this study are listed in Table 1. relative expression was analyzed by the 2-ΔΔCt method (Livak method). The data for this study can be obtained by downloading the Supplementary File.

### Statistical analysis

The experimental data were expressed as mean ±standard deviation, differences between the two groups were analyzed using *t*-test, and differences between the three groups were analyzed using analysis of variance (ANOVA). GEO data were transformed from ENTRZ ID annotation to SYMBOL ID by R language, and after voom normalization by limma (Ritchie et al., 2015), differential genes were screened and GO enrichment analysis and KEGG pathway enrichment analysis were performed using clusterProfiler (Yu et al., 2012). Differentially expressed mRNA clustering analysis was performed with FC values using the heatmap function of R. Based on the ploidy change log(FC) ≥ 1.0 and P.value < 0.05 as the differential screening threshold, and based on this, Gene Ontology (GO) and Kyoto Encyclopedia of Genes and Genomes (KEGG) enrichment analyses were performed.GO analysis included biological processes, cellular components, and molecular functions; KEGG pathway analysis inferred the biological signaling pathways they might be involved in, with p .value< 0.05 as the threshold for significant enrichment.

**Table 1:** List of 12 gene primers.

**Genes**	**5′–3′**	**Primer sequences**
Cd74	Forward Reverse	CATGGATGACCAACGCGAC TGTACAGAGCTCCACGGCTG
H2-Aa	Forward Reverse	TCAGTCGCAGACGGTGTTTAT GGGGGCTGGAATCTCAGGT
Ccl5	Forward Reverse	AGATCTCTGCAGCTGCCCTCA GGAGCACTTGCTGCTGGTGTAG
H2-DMa	Forward Reverse	CAAGCTTCTCCTCAGCGACT CCTTCCAGATCCATCACGTTT
Psmb9	Forward Reverse	CGTGAGGACTTGTTAGCGCA CTCACATTGGTCCCAGCCA
Psmb8	Forward Reverse	GAAGACGGTTGGGTGAAAGT ACTGAAGTAGTCCCAGGTCTC
Cd3g	Forward Reverse	TCTCTACTGGGCTCTCTCCAA CCATCTCCAAGGAAACCAAC
Ubd	Forward Reverse	CTCTGGTTTCTGGCCCCTTG ATTCCTCGGAACGGACATGC
Gzmb	Forward Reverse	GCTGCTCACTGTGAAGGAAGT TGGGGAATGCATTTTACCAT
Saa2	Forward Reverse	GACACCAGCAGGATGAAG CCAACACAGCCTTCTGAACT
Saa1	Forward Reverse	GAGGACATGAGGACACCATTGC CCAGAGAGCATCTTCAGTGTTCC
Saa3	Forward Reverse	CAGGATGAAGCCTTCCATTG CATGACTGGGAACACAGGA

## Results

### Low-dose penicillin affects the immune pathway of the Ileum in mice

**Figure 2:**
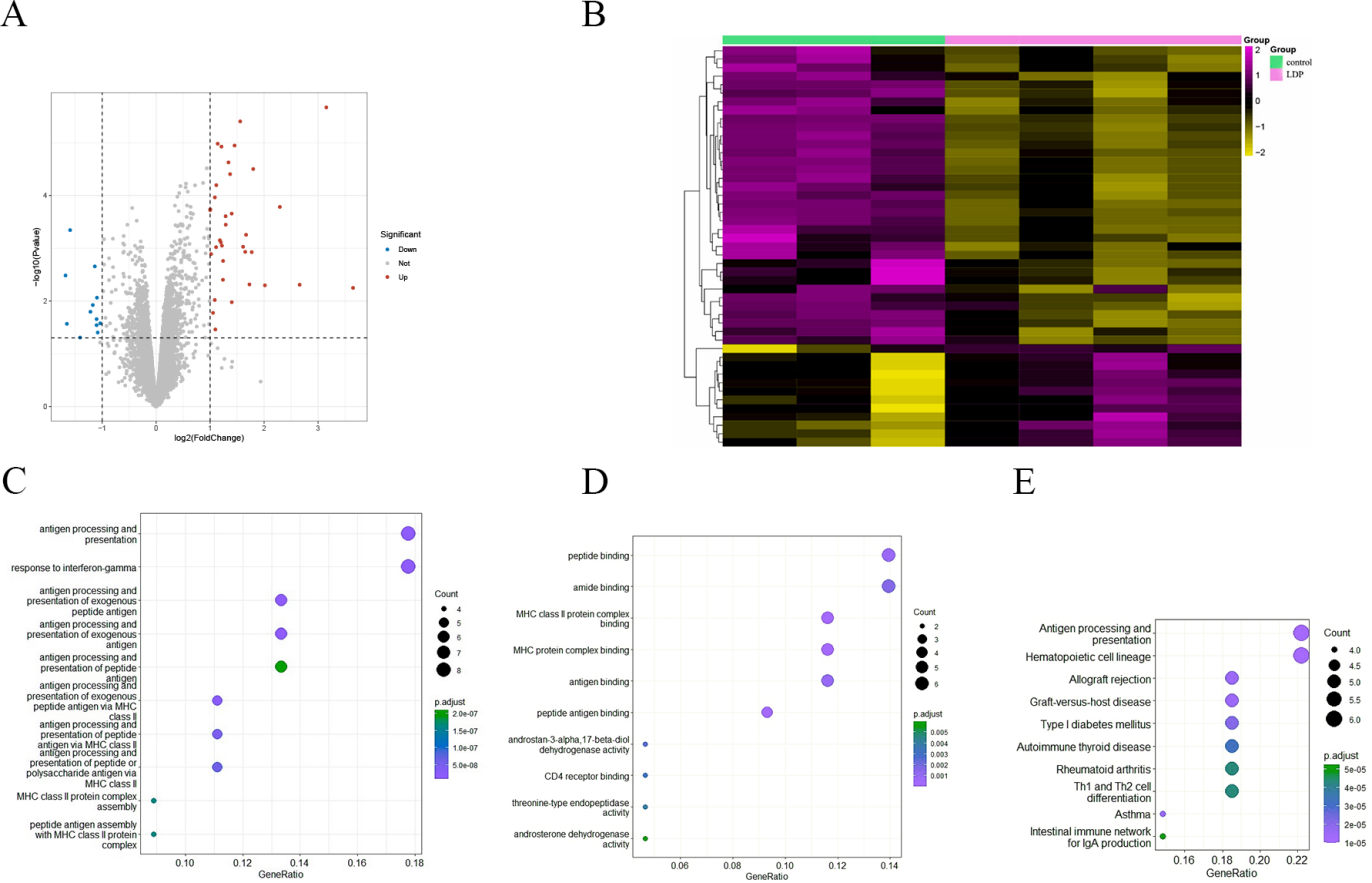
(A) Volcano diagram of all genes. Blue indicates down-regulated genes and red indicates up-regulated genes. (B) Hierarchical clustering of differential genes. Each column represents a sample and each row represents a gene. High and low expression are indicated in red and green, respectively. (C–D) Differential gene GO (BP and MF) enrichment. The circle size represents the number and the circle color represents the *P* value. The horizontal coordinate is the gene frequency and the vertical coordinate is the reaction name (E) KEGG enrichment. Horizontal coordinates are gene frequencies, vertical coordinates are pathway names.

To investigate the effect of antibiotics on intestinal gene expression, the GSE58087 microarray dataset was selected for analysis. Four male mice were exposed to low-dose penicillin (LDP) (6.7 mg/L) from birth and three mice were born normally as control, ileum was collected at eight weeks of age, RNA was extracted, and the differences were analyzed, with —FC—>1 representing significant and *P* value < 0.05 representing statistically significant screening for differential genes. As shown in the volcano plot in Fig. 2A, a total of 35 up-regulated genes *vs.* 12 down-regulated genes, and was plotted by hierarchical clustering (see Fig. 2B). Each column represents the expression pattern of a sample; high and low expression levels are indicated by red and green lines, respectively, with a clear distribution of differential genes. GO enrichment analysis of differential genes, in biological processes, as in Fig. 2C, is mainly involved in antibody processing and presentation, interferon IFN-γ response, exogenous peptide antibody processing and presentation, and peptide antibody processing and presentation *via* MHC-II. In the molecular function, as in Fig. 2D, the peptide and amide binding, MHC-II protein complex binding, and antibody and peptide antibody binding processes are mainly carried out. Antigen presentation as a bridge between natural and acquired immunity. Antigen processing is the process by which cells *in vivo* collect antigens and degrade them into peptides, while antigen presentation is the process by which processed antigen peptides are presented to the cell surface for easy recognition by T cells. MHC can bind to peptides to form protein complexes and provide feedback to T cells by presenting antigenic peptides. MHC-II mainly presents exogenous peptides (*i.e.,* from extracellular sources), is recognized by CD4+ T cells (helper T cells), is expressed only on the surface of antigen-presenting cells such as macrophages, dendritic cells, and B cells, and is known to be involved primarily in the immune response. Low-dose penicillin alters the expression of immune-related genes in mice and disrupts the original balance of immune gene expression in the mouse ileum. A KEGG pathway enrichment analysis was then performed, as shown in Fig. 2E. It was mainly enriched to antibody processing and presentation, hematopoietic cell lineage, allogeneic rejection, graft-versus-host disease, and TH1 and TH2 cell differentiation pathways, highly suggesting that it is closely related to the immune system. This again demonstrates the involvement of the intestinal microbiota in numerous immune-like pathways.

**Figure 3:**
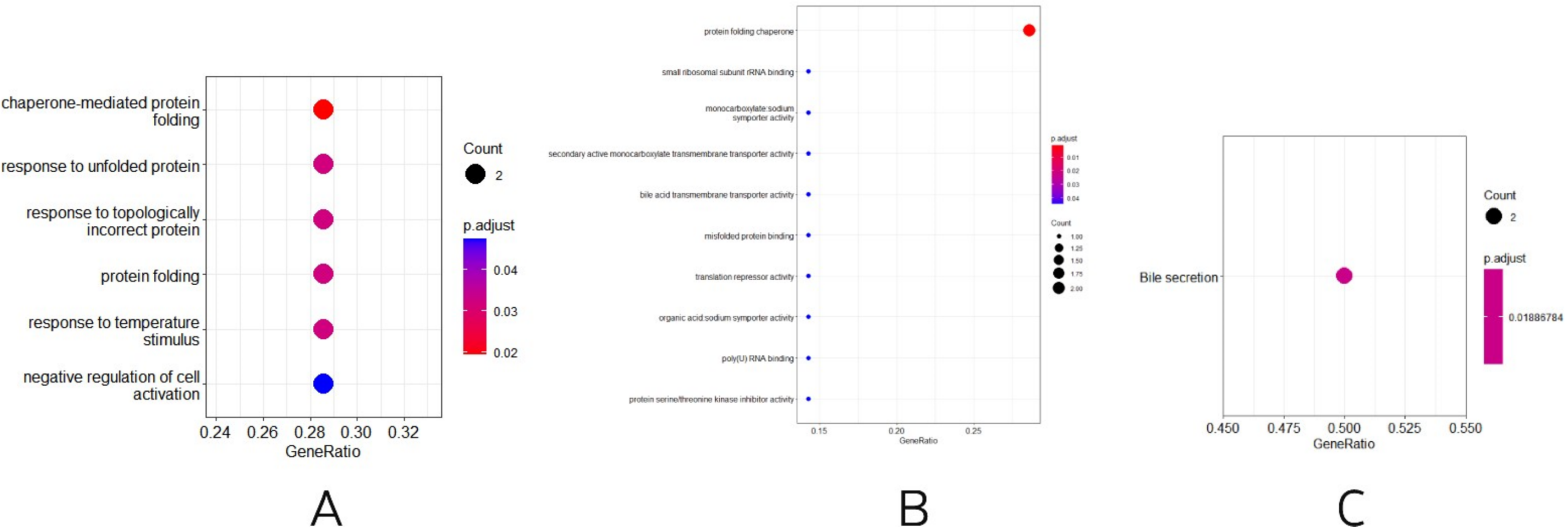
(A–B) Differential gene GO (BP and MF) enrichment. The circle size represents the number and the circle color represents the *P* value. Horizontal coordinates are gene frequencies, vertical coordinates are reaction names. (C) KEGG enrichment. Horizontal coordinates are gene frequencies, vertical coordinates are pathway names.

Three other normally grown mice and three mice receiving low penicillin injections had their livers collected at 30 weeks of age and RNA extracted. differential analysis revealed three upregulated genes, Neat1, Cirbp, and Rian, and five downregulated genes, Hspa1b, Ugt2b38, Hspb1, Cidec, and Slc10a2 (Table 2). Enrichment analysis of differential genes, in biological processes, as in Fig. 3A, were mainly involved in chaperone-mediated protein folding, response to unfolded proteins, response to topologically incorrect proteins, protein folding, response to temperature stimulation, negative regulation of cell activation response, associated with protein folding, temperature stimulation, and cell activation. In the molecular functions, as in Fig. 3B, the protein folding chaperones were mainly involved. Then KEGG pathway enrichment analysis was performed, as in Fig. 3C, enriching the pathway of bile secretion.

**Table 2:** List of differential genes between liver RNA low penicillin and normal groups in 30-week-old mice.

**Genes**	**Orthologous (human)**	**Pathways**
Cd74	CD74	NF-kappaB Signaling, Class I MHC mediated antigen processing and presentation, Cell surface interactions at the vascular wall, Response to elevated platelet cytosolic Ca2+, Innate Immune System
H2-Aa	HLA-DQA1, HLA-DQA2	TCR Signaling (Qiagen), TCR signaling (REACTOME), Phosphorylation of CD3 and TCR zeta chains, Immune response Antigen presentation by MHC class II, Immune response NFAT in immune response
Ccl5	CCL5	MIF Mediated Glucocorticoid Regulation, TGF-Beta Pathway, Akt Signaling, CCR5 Pathway in Macrophages, ERK Signaling
H2-DMa	HLA-DMA	TCR Signaling (Qiagen), Immune response Antigen presentation by MHC class II, Immune response NFAT in immune response, CD28 Signaling in T-Helper Cell, Immune response Function of MEF2 in T lymphocytes
Psmb9 Psmb8	PSMB9 PSMB8	Regulation of activated PAK-2p34 by proteasome mediated degradation, Assembly of the pre-replicative complex, Chks in Checkpoint Regulation, Class I MHC mediated antigen processing and presentation, RAF/MAP kinase cascade
Cd3g	CD3G	Regulation of actin dynamics for phagocytic cup formation, Immune response NFAT in immune response, TCR Signaling (Qiagen), ADORA2B mediated anti-inflammatory cytokines production, TCR signaling (REACTOME)
Ubd	UBD	BRCA1 Pathway, Metabolism of proteins, NF-kappaB Signaling, Beta-Adrenergic Signaling, HIF1Alpha Pathway
Gzmb	GZMB	Granzyme Pathway, Intrinsic Pathway for Apoptosis, Programmed Cell Death, NOTCH2 Activation and Transmission of Signal to the Nucleus, Regulated Necrosis
Saa2 Saa1 Saa3	SAA1 SAA2 SAA3P	TRAF6 mediated induction of NFkB and MAP kinases upon TLR7/8 or 9 activation, GPCR downstream signalling, Class A/1 (Rhodopsin-like receptors), Binding and Uptake of Ligands by Scavenger Receptors, Cytokine Signaling in Immune system

### Identification of 12 key genes

Through literature searches and related websites, 12 key genes in the differential genes were selected for validation: Cd74, H2-Aa, Ccl5, H2-DMa, Psmb9, Psmb8, Cd3g, Ubd, Gzmb, Saa2, Saa1, Saa3, genes mostly enriched in signaling pathways of immunity and inflammation (Table 3). CD74 is an invariant chain expressed on the plasma membrane and presents an evolutionarily conserved type II membrane protein, which is involved in many different activities and is a key gene in both immune and inflammatory signaling pathways (Borghese & Clanchy, 2011). Major histocompatibility complex class II (MHC II) is an important immune regulatory molecule that plays an important role in both antigen presentation and immune cell development. H2-Aa, as part of the MHC class II protein complex, has been associated with a variety of diseases, including autoimmune diseases and cancers of the gastrointestinal system, and is a significant pro-inflammatory-related gene (Zhao et al., 2022). Ccl5 is homologous to human CCL5 (C-C motif chemokine ligand 5), a key pro-inflammatory chemokine that induces *in vitro* migration and recruitment of a variety of cells, including T cells, and plays a role in a variety of biological processes including cancer and atherosclerosis. ccl5 also interacts with a variety of other receptors to regulate cellular activity, receptor and chemokine levels and maintain homeostasis in vivo (Lapteva & Huang, 2010). H2-DMa facilitates the exchange of peptides bound to MHC class II molecules and plays an important role in humoral immune responses, but different class II molecules have different sensitivities to it (Alfonso et al., 2001). Psmb9 and Psmb8 are genes encoding immunoproteasome subunits that act upstream or within antigen processing, bacterial presentation, and response, and have been implicated in both lymphoma and renal cell carcinoma. CD3G is involved in the formation of the TCR-CD3 complex and immune response, and its mutation often leads to diseases such as inflammation and cancer (Birnbaum et al., 2014). Ubiquitin D (UBD) is a member of the ubiquitin-like (UBL) modifier family, highly expressed in a variety of cancers including colorectal cancer (CRC) (Su et al., 2021), and is also involved in cellular protein metabolic processes; positive regulation of I-kappaB kinase/NF-kappaB signaling; and positive regulation of apoptotic processes. It acts upstream or internally in response to interferon-gamma and response to tumor necrosis factor, located in the cytoplasm and nucleus and expressed in the digestive system, aorta, male gonads or organs, olfactory lobes, and thymus. GZMB encodes a member of the granzyme subfamily of proteins and is part of the S1 family of serine proteases. It has a positive role in the clearance of tumor cells, bacteria, parasites, and viruses, as well as in the recognition, diagnosis, and treatment of diseases, but have a negative role in extracellular functions (Wang et al., 2021). The acute phase members of the mouse serum amyloid A (SAA) family, Saa1, Saa2, and Saa3, are highly similar at both the nucleotide and protein sequence levels, but respond differently to different pro-inflammatory cytokines (Thorn & Whitehead, 2002), and SAA3 was found to be pro-atherogenic (Thompson et al., 2018).

**Table 3:** Human orthologous of 12 genes and related signaling pathways.

**Genes**	**Orthologous (human)**	**Pathways**
Cd74	CD74	NF-kappaB Signaling, Class I MHC mediated antigen processing and presentation, Cell surface interactions at the vascular wall, Response to elevated platelet cytosolic Ca2+, Innate Immune System
H2-Aa	HLA-DQA1, HLA-DQA2	TCR Signaling (Qiagen), TCR signaling (REACTOME), Phosphorylation of CD3 and TCR zeta chains, Immune response Antigen presentation by MHC class II, Immune response NFAT in immune response
Ccl5	CCL5	MIF Mediated Glucocorticoid Regulation, TGF-Beta Pathway, Akt Signaling, CCR5 Pathway in Macrophages, ERK Signaling
H2-DMa	HLA-DMA	TCR Signaling (Qiagen), Immune response Antigen presentation by MHC class II, Immune response NFAT in immune response, CD28 Signaling in T-Helper Cell, Immune response Function of MEF2 in T lymphocytes
Psmb9 Psmb8	PSMB9 PSMB8	Regulation of activated PAK-2p34 by proteasome mediated degradation, Assembly of the pre-replicative complex, Chks in Checkpoint Regulation, Class I MHC mediated antigen processing and presentation, RAF/MAP kinase cascade
Cd3g	CD3G	Regulation of actin dynamics for phagocytic cup formation, Immune response NFAT in immune response, TCR Signaling (Qiagen), ADORA2B mediated anti-inflammatory cytokines production, TCR signaling (REACTOME)
Ubd	UBD	BRCA1 Pathway, Metabolism of proteins, NF-kappaB Signaling, Beta-Adrenergic Signaling, HIF1Alpha Pathway
Gzmb	GZMB	Granzyme Pathway, Intrinsic Pathway for Apoptosis, Programmed Cell Death, NOTCH2 Activation and Transmission of Signal to the Nucleus, Regulated Necrosis
Saa2 Saa1 Saa3	SAA1 SAA2 SAA3P	TRAF6 mediated induction of NFkB and MAP kinases upon TLR7/8 or 9 activation, GPCR downstream signalling, Class A/1 (Rhodopsin-like receptors), Binding and Uptake of Ligands by Scavenger Receptors, Cytokine Signaling in Immune system

### Differential expression of key genes in the intestine and liver after antibiotic treatment

**Figure 4:**
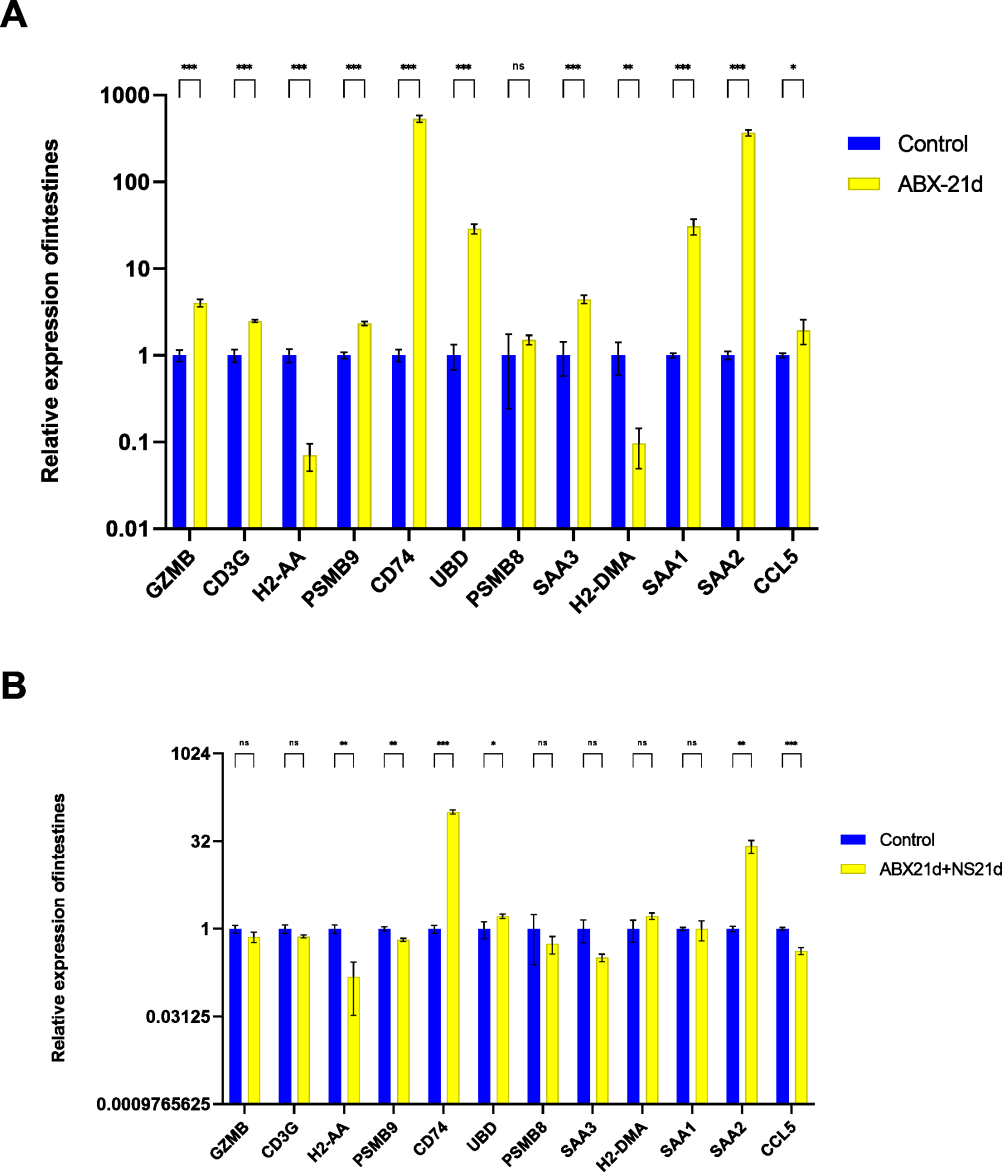
Relative expression of key genes in the intestine. (A) Blank control *vs* antibiotic treatment for 21 days. (B) Blank control *vs* antibiotic treatment for 21 days followed by saline feeding for 21 days. (*T*-test significance marker: ns: q> =0.05, *: 0.01 ****: q< =0.0001).

**Figure 5:**
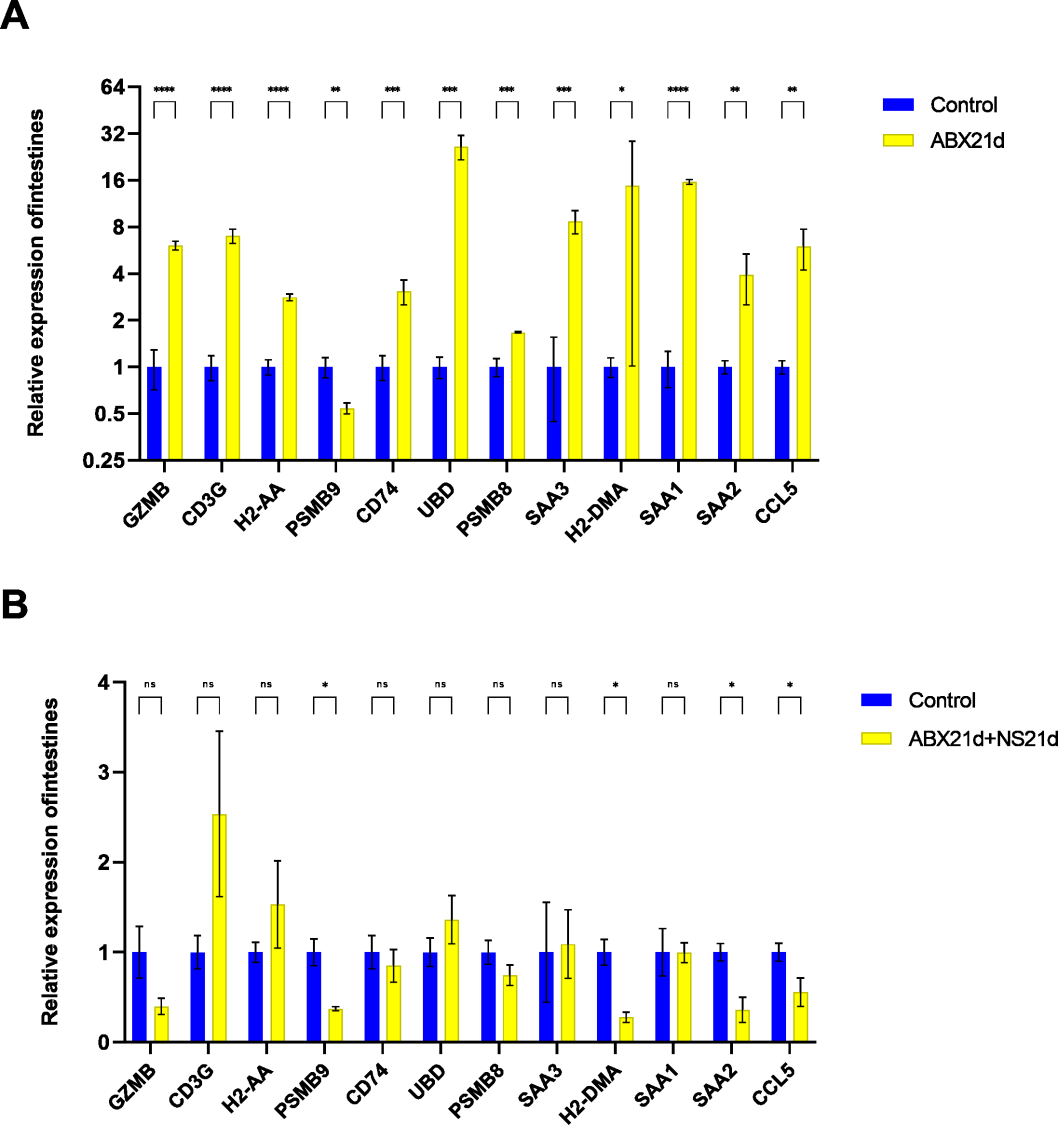
Relative expression of key genes in the liver. (A) Blank control *vs* antibiotic treatment for 21 days. (B) Blank control *vs* antibiotic treatment for 21 days followed by saline feeding for 21 days. (*T*-test significance marker: ns: q> =0.05, *: , **: 0.001¡q¡ =0.01, ***: 0.0001¡q¡ =0.001, ****: q¡ =0.0001).

To verify whether different antibiotics would affect gene expression, we treated mice with neomycin and ampicillin for 21 days, then recovered naturally for 21 days, and then examined their relative expression to the normal group by qPCR. Mice treated with antibiotics for 21 days showed significantly elevated expression of 11 genes in the intestine, except for H2-DMa, and most of them were significantly different. After 21 days of normal saline feeding, *i.e.,* natural recovery, GZMB, CD3G, PSMB8, SAA3, H2-DMA, and SAA1 all returned to about normal levels, but H2-AA, PSMB9, CD74, UBD, SAA2, and CCL5 still had expression differences, with H2-AA, PSMB9, and CCL5 showing reduced expression and CD74, UBD, and SAA2 expression was elevated (Fig. 4, Tables S1, S2). The same expression trend remained in the liver, with significantly higher expression except for PSMB9, but the increase was not as high as in the intestine, and most genes also returned to normal levels after natural recovery, with only H2-DMA, SAA2, CCL5, and PSMB9 remaining significantly lower (Fig. 5, Tables S3, S4).

### Fecal microbiota transplantation in healthy mice may have additional effects on key gene expression

**Figure 6:**
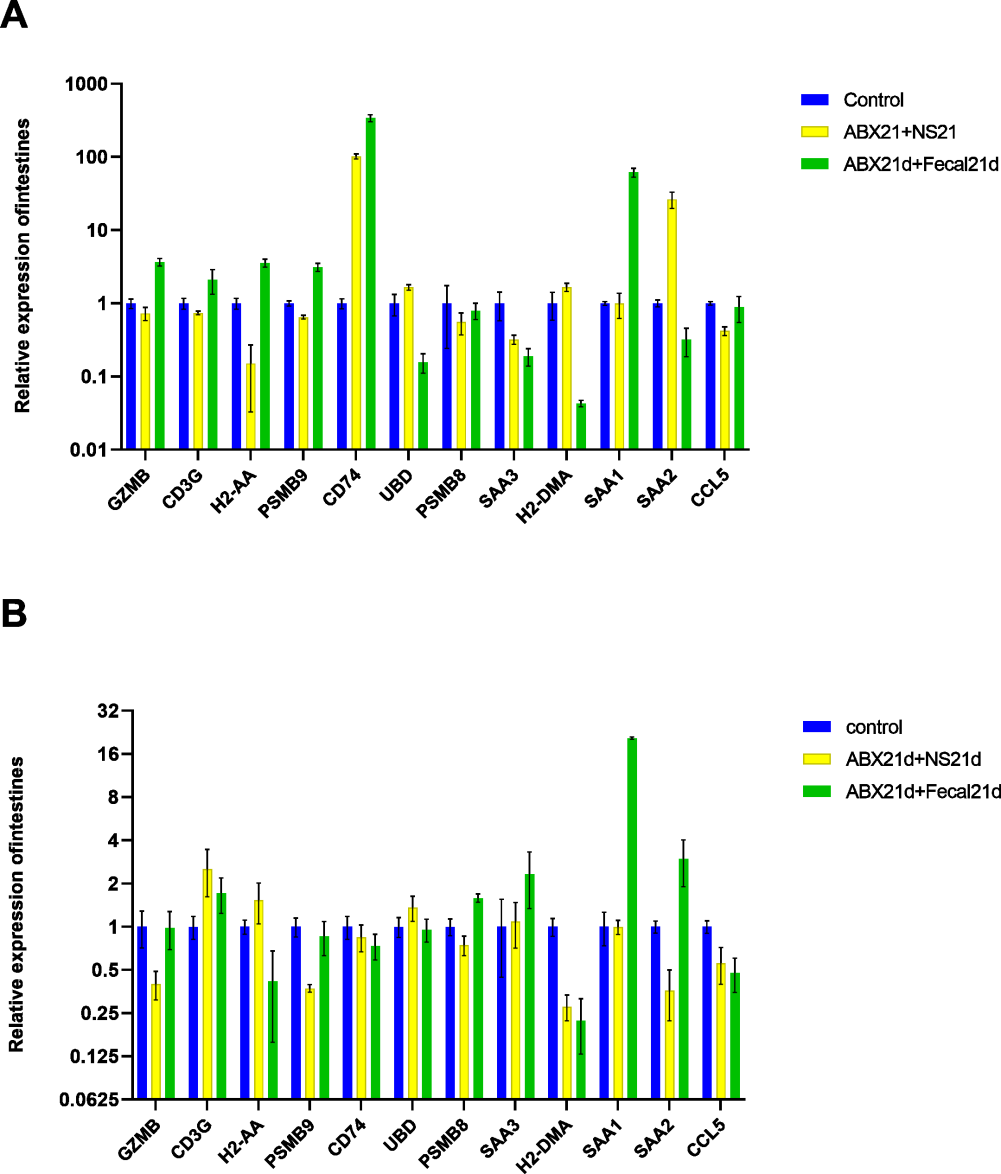
(A) Relative expression of key genes in the intestine. blank control *vs* antibiotic treatment for 21 days (NS) *vs* antibiotic treatment for 21 days after fecal microbiota trans-plantation for 21 days (FMT). (B) Relative expression of key genes in the liver, blank control *vs* antibiotic treatment for 21 days (NS) *vs* antibiotic treatment for 21 days after fecal microbiota transplantation for 21 days (FMT). (ANOVA significance marker: ns: p¿ =0.05, *: 0.01¡p¡0.05, **: 0.001¡p¡ =0.01, ***: 0.0001¡p¡ =0.001, ****: p¡ =0.0001).

To determine whether fecal microbiota transplantation after antibiotic treatment facilitates the re-establishment of intestinal microbiota and the effect on intestinal *versus* liver genes, we continued FMT for 21 days in experimental groups of mice treated with antibiotics for 21 consecutive days. The qPCR results showed that FMT did not help to restore genes to normal levels in intestinal or liver tissues, but in intestinal tissues, genes that were restored more normally in the NS group (also known as the natural recovery group) were highly expressed in the FMT group, with only SAA2 significantly lowly expressed, CD74 still highly expressed, and UBD largely unexpressed. For liver tissues, the expression of H2-DMA remained low, the expression of SAA1, SAA2, SAA3, and PSMB8 was significantly higher, H2-AA was significantly lower, and only GZMB, CD3G, and PSMB9 changed in a good direction (Fig. 6, Table S5).

### Vitamin C contributes to the restoration of gene expression in intestinal and liver tissues

Studies have shown that specific dietary interventions and micronutrient modulation of intestinal microbes are positive therapeutic strategies, while vitamin C (ascorbic acid) has been shown to have health benefits and to be involved in biological processes that support the immune system (Carr & Maggini, 2017). Vitamin C supplementation also has a protective effect against oxidative stress and has received attention for its role in oxidative stress-related pathological diseases. Studies have confirmed that supplementation with high doses of vitamin C can manipulate the composition of the intestinal microbiota, thereby altering the intestinal microbiota (Otten et al., 2021).

**Figure 7:**
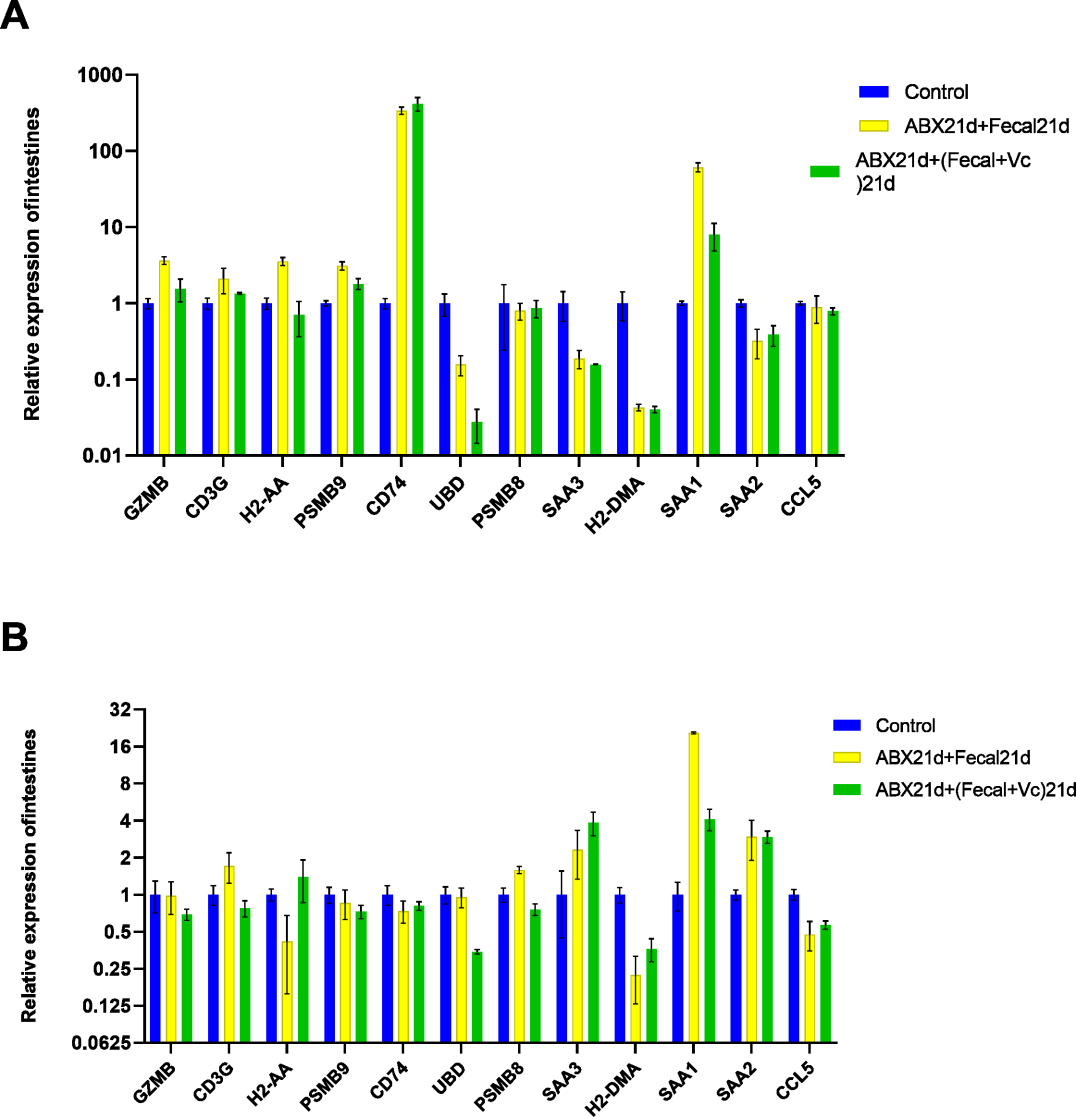
(A) Relative expression of key genes in the intestine. blank control *vs* 21 days of antibiotic treatment followed by fecal transplantation for 21 days GSE580587 21 days of antibiotic treatment followed by fecal transplantation plus vitamin C. (B) Relative expression of key genes in the liver. Blank control GSE580587 antibiotic treatment for 21 days after fecal transplantation for 21 days GSE580587 antibiotic treatment for 21 days after fecal transplantation plus vitamin C for 21 days. (ANOVA significance marker: ns: p¿ =0.05, *: 0.01 < *p* < 0.05, **: 0.001¡p¡ =0.01, ***: 0.0001¡p¡ =0.001, ****: p¡ =0.0001).

To investigate whether vitamin C could affect fecal microbiota transplantation, we designed an experimental group in which mice were transplanted with fecal microbes for 21 consecutive days after 21 days of Abx treatment and given the same dose of vitamin C. The qPCR results showed that for intestinal tissues, GZMB, CD3G, H2-AA, PSMB9, SAA1 had better results and were closer to the genes in the normal group, reducing the expression of their highly expressed genes in the F group, these genes function in Granzyme Pathway, Intrinsic Pathway for Apoptosis, Programmed Cell Death, Immune response NFAT in immune response, TCR Signaling (Qiagen), Class I MHC mediated antigen processing and presentation, Cytokine Signaling in Immune system, among other pathways. The addition of vitamin C had no significant effect on CD74, UBD, PSMB8, SAA3, H2-DMA, SAA2, and CCL5, but it did not cause worse effects either. In liver tissues, more favorable effects were found for GZMB, CD3G, H2-AA, PSMB8, SAA1, and less favorable effects for the other seven genes. It is also important to note that the expression of genes in both tissues was higher in the intestine than in the liver for group F genes, and the same was true after the addition of vitamin C. This suggests that vitamin C acts directly in the intestine and more weakly in the liver. In conclusion, for intestinal tissues, the introduction of vitamin C restored the genes more to normal levels, and for liver tissues, vitamin C mostly reduced the expression of highly expressed genes (Fig. 7, Table S5).

## Discussion

Intestinal microbes are microorganisms that reside in the intestinal and play an important role in the development of metabolic diseases, cancer, and neurological disorders, and a large number of factors have an impact on the number and diversity of intestinal microbes, such as lifestyle, dietary intake, and medications (Qi et al., 2021). Recent studies have shown that the global use of antibiotics in human medicine is on the rise. However, incorrect antibiotic prescriptions still exist; it is estimated that as many as half of the people take antimicrobials unnecessarily (Bassetti et al., 2022). The response of microbiota to antibiotics varies from person to person, and the mechanisms of response are unknown. It has been shown that intestinal microbiota plays a role in the pathogenesis of inflammatory bowel disease and that antibiotics play a role in the treatment of inflammatory bowel disease (Perencevich & Burakoff, 2006); similarly, the intestinal microbiota mediates the development of hepatocellular carcinoma, bile acids promote the release of inflammatory cytokines through multiple pathways, and bacterial toxins from the intestinal microbiota can damage DNA indirectly through ROS, or directly by damaging host DNA. (Luo et al., 2022).

In this study, we investigated the effect of antibiotics on the differential expression of relevant immune-inflammatory genes in the intestine and liver. First, we screened the ileal *versus* liver differentially expressed genes under the influence of low-dose penicillin in the GSE58087 microarray dataset. The differential genes were found to be mostly enriched in immune responses such as antibody treatment and expression, interferon IFN-γ response, and exogenous peptide antibody treatment and expression in intestinal tissues, suggesting that antibiotics mediate the occurrence of intestinal immune responses through intestinal microbes. However, the nine differential genes identified in liver tissue did not overlap with those in ileal tissue: Neat1, Cirbp, Rian/Meg8, Hspa1b, Ugt2b38, Hspb1, Cidec, and Slc10a2, and were mainly enriched in protein folding. This may be due to the direct effect of antibiotics on the intestine and their impact on the intestinal flora, which in turn indirectly affects the liver through the ‘enterohepatic axis’. We mainly investigated the changes in immune genes after antibiotic-induced inflammation and the subsequent recovery, so we selected 12 key genes for subsequent validation analysis after reviewing the literature and related literature.

In intestinal tissues, all 10 genes were significantly overexpressed, except H2-AA with H2-DMA. After natural recovery by normal saline gavage, H2-AA remained lowly expressed, CD74 and SAA2 remained highly expressed but largely decreased gradually, while the other genes largely returned to normal levels. It is suggested that antibiotics activate various inflammatory signals by affecting intestinal microbes. CD74 differs most from SAA2. CD74 is an immune response-related transcription factor involved in the inflammatory response and hematopoietic development and function, and activation of CD74 causes transcriptional activation, while the genes it regulates include genes of the NF-κB pathway and cell death, genes related to immune cell motility and leukocyte recruitment, and genes for blood cell interactions and adhesion are all rapidly upregulated when it is activated (Gil-Yarom et al., 2017). Circulating serum amyloid A2 (SAA2) is increased under inflammatory conditions and is a marker of inflammation that also activates a large number of immune cells (De Buck et al., 2016). So antibiotics cause damage to the organism after regulating intestinal microorganisms or after infection, activating two factors, CD74 and SAA2, and activating a large number of genes and cells at the same time. For liver tissues, all genes except PSMB9 were also upregulated in expression and downregulated after natural recovery, while PSMB9 remained lowly expressed. The proteasome *β*9 subunit plays an important role in the human leukocyte antigen class I (HLAI) delivery system (Li et al., 2020), suggesting that antibiotics may have inhibited the expression of PSMB9 on antigen delivery.

To further confirm the effect of intestinal microbes on gene expression, we attempted to restore the microbiota of mice by fecal microbiota transplantation after ABX treatment of mice. A study showed that in mice treated with FMT after antibiotics and chemotherapy, dysregulation of the intestinal microbiota was rapidly corrected and no significant pathogenic bacteria appeared in the intestine. Thus, FMT restores a healthy and diverse intestinal microbiota that may potentially prevent the emergence of pathogenic bacteria and bacterial translocation from the gastrointestinal tract to the bloodstream (Berg, 1999). However, our study found that genes were not restored to normal levels after fecal transplantation, which may be due to irreversible damage to the intestinal and liver caused by changes in the microbiota, or it may be that strain incompatibility between FMT donors and recipients prevented microbiome transplantation or that the host immune response to the transplanted microbiota may lead to FMT rejection. Among them, compared with the NS group, genes in the FMT group were highly expressed in intestinal tissues, GZMB, CD3G, H2-AA, PSMB9, CD74, SAA1, indicating that FMT might bring some inflammatory response, but SAA2 was down-regulated, and there was no significant difference with the normal group, then FMT also reduced the expression of SAA2 to some extent. In liver tissues, several genes in the FMT group were not significantly different from the NS group, but SAA1, SAA2, and SAA3 were all highly expressed, which may be because SAA1 and SAA2 are synthesized and secreted in the liver. And SAA is secreted after hepatocytes are stimulated by pro-inflammatory cytokines or tumor necrosis factors (Thorn, Lu & Whitehead, 2004). It suggests that FMT stimulated chemokines of immune cells in the liver and upregulated expression of several inflammatory factors, but the effect of this condition on the organism is not yet known.

Vitamin C plays an important role in several ways; it is a potent major non-enzymatic antioxidant in plasma that reacts with and scavenges free radicals, thereby inhibiting chain reactions and thus preventing lipid peroxidation damage (Frei, Stocker & Ames, 1988), Vitamin C reversed the increased oxidative stress in this cellular compartment due to the mislocalization of Wrn mutant protein in the endoplasmic reticulum fraction of the liver (Aumailley et al., 2015). Similarly, vitamin C has been identified as a major physiological antioxidant in more developed organisms and has anti-inflammatory properties (Padayatty et al., 2003). We tried to investigate whether vitamin C could have a positive effect on fecal microbial transplantation by adding vitamin C to fecal microbial transplantation. In intestinal tissues, the expression of genes that were highly expressed after fecal microbiota transplantation was found to be effectively reduced, while normally expressed genes were not affected, but CD74 genes remained highly expressed, suggesting that the addition of vitamins did not affect the immune response in the intestine. In liver tissue, normally expressed genes were also unaffected, but the expression of SAA1 was significantly reduced, while the expression of SAA3 was elevated, and it has been shown that elevated SAA3 protects the colonic epithelium from acute injury through TLR2-dependent induction of neutrophil IL-22 expression in a mouse model of colitis (Zhang et al., 2018). That is, the addition of vitamin C effectively reduced the high gene expression due to fecal microbial transplantation and ameliorated the possible bad effects of FMT, regulating the homeostatic balance of the immune system, and was more effective in the intestine and weakened in the liver. Its role can be continued to be explored in future studies to provide a layer of assurance for fecal microbial transplantation.

## Conclusions

In this study we determined that antibiotics mediated gene expression in the intestinal and liver by modulating intestinal microbes; therefore, incorporating the intestinal and liver response is needed when using antibiotics in the future. We also found that fecal microbial transplantation did not necessarily have a positive effect on intestinal microbiota recovery, but the addition of vitamin C did have a positive effect, but more factors still need to be explored. Again this study has some limitations, the selection number of genes is too small and does not reflect the complete changes in the intestinal and liver. There was no sampling at multiple time points, and it is not particularly clear how deeply the intestinal microbiota affects the genes. Due to experimental errors, two extreme data were removed when conducting the mice experiments, resulting in only three mice per group and reduced reliability of the experiment. In future studies, we hope to improve the process and content.

##  Supplemental Information

10.7717/peerj.15356/supp-1 Supplemental Information 1 Changes in gene expression in the intestine by qPCR

10.7717/peerj.15356/supp-2 Supplemental Information 2 Changes in gene expression in the liver by qPCR

10.7717/peerj.15356/supp-3 Supplemental Information 3 Multiple unpaired t tests of Figure 4A

10.7717/peerj.15356/supp-4 Supplemental Information 4 Multiple unpaired t tests of Figure 4B

10.7717/peerj.15356/supp-5 Supplemental Information 5 Multiple unpaired t tests of Figure 5A

10.7717/peerj.15356/supp-6 Supplemental Information 6 Multiple unpaired t tests of Figure 5B

10.7717/peerj.15356/supp-7 Supplemental Information 7 ANOVA results for Figures 6 and 7

10.7717/peerj.15356/supp-8 Supplemental Information 8 Author Checklist

## Additional Information and Declarations

### Data Availability

The following information was supplied regarding data availability: The raw measurements are available in the Supplemental Files.
